# Draft Genome Sequence of *Salmonella enterica* subsp. *enterica* Serovar Widemarsh Strain CRJJGF_00058 (Phylum *Gammaproteobacteria*)

**DOI:** 10.1128/genomeA.00604-16

**Published:** 2016-07-14

**Authors:** Sushim K. Gupta, Elizabeth A. McMillan, Charlene R. Jackson, Prerak T. Desai, Steffen Porwollik, Michael McClelland, Lari M. Hiott, Shaheen B. Humayoun, Jonathan G. Frye

**Affiliations:** a Bacterial Epidemiology and Antimicrobial Resistance Research Unit, U.S. National Poultry Research Center, U.S. Department of Agriculture, Agricultural Research Service, Athens, Georgia, USA; b Department of Microbiology, University of Georgia, Athens, Georgia, USA; c Department of Microbiology and Molecular Genetics, University of California Irvine, Irvine, California, USA

## Abstract

Here, we report a 4.73 Mbp draft genome sequence of *Salmonella enterica* subsp. *enterica* serovar Widemarsh strain CRJJGF_00058, isolated from eggs in 2008.

## GENOME ANNOUNCEMENT

The presence of *Salmonella* in eggs is a potential threat to public health. Egg laying hens are one of the leading sources of *Salmonella* outbreaks (1). The rarely reported *Salmonella* serovar Widemarsh has been isolated from pasteurized dried egg white, however, this serovar was not listed in the USDA FSIS list of most common *Salmonella* isolated from liquid egg product (2).

The *Salmonella* strain CRJJGF_00058 was isolated from eggs in 2008 using standard microbiology techniques and serotyped using SMART typing (3). The isolate was serogrouped using serogroup-specific antisera (Difco Laboratories, Detroit, MI) and the serovar was confirmed at the National Veterinary Services Laboratories, APHIS, USDA (Ames, IA). This bacterium belonged to antigenic group O:35(O), along with *Salmonella* serovar IIIa, and exhibited only phase 1 flagellar H antigens z29 (35:z29:-) (4). MICs (µg/ml) were determined by broth microdilution using the Sensititre semi-automated antimicrobial susceptibility system (TREK Diagnostics Systems, Thermo Fisher Scientific, Inc., Oakwood Village, OH). Results were interpreted according to the Clinical and Laboratory Standards Institute (CLSI) guidelines (5). The strain was susceptible to all tested antibiotics. However, we detected a cryptic aminoglycoside resistance gene (*aac6-Iy*) in the genome (6).

The genomic DNA was isolated using a GenElute bacterial genomic DNA kit (Sigma-Aldrich, St. Louis, MO) and the DNA library was constructed using a Nextera-XT DNA preparation kit and paired-end sequencing was performed on an Illumina HiSeq2500 (Illumina Inc., San Diego, CA) using a 500-cycle MiSeq reagent kit. About 3,743,028 reads with quality score >30 were assembled using Velvet assembler (7), which resulted in 164 contigs with minimum contig length ≥200 bp. The total assembly size was 4.73 Mbp, with *N*_50_ values of 66.7 kb, and G+C content of 52.06%. The generated contigs were ordered with MAUVE using *Salmonella* LT2 as a reference (8). Genes were predicted with prodigal (9) and ARAGORN (10) was used to predict tRNAs. A total of 4,401 coding sequences (≥50 amino acids) and 45 tRNAs were predicted within the genome. Prophages, clustered regularly interspaced short palindromic repeats (CRISPR), and signal peptides were predicted using PHAST (11), CRISPRFinder (12), and signalp (13), respectively. We identified signal peptides in 445 genes, three CRISPR loci, and 1-intact/2-incomplete phages in the genome. Addition of this rarely reported *Salmonella* Widemarsh genome will improve our understanding of the genetics and pathogenicity of *Salmonella* serovars.

### Nucleotide sequence accession number.

Genome sequences of *Salmonella enterica* subsp. *enterica* serovar Widemarsh strain CRJJGF_00058 have been deposited in GenBank under the accession number JQUO00000000. This paper describes the first version of the genome.
